# Longitudinal Alzheimer’s Disease Progression Prediction With Modality Uncertainty and Optimization of Information Flow

**DOI:** 10.1109/JBHI.2024.3472462

**Published:** 2025-01-07

**Authors:** Duy-Phuong Dao, Hyung-Jeong Yang, Jahae Kim, Ngoc-Huynh Ho

**Affiliations:** Department of Artificial Intelligence Convergence, Chonnam National University, Gwangju 61186, South Korea; Department of Artificial Intelligence Convergence, Chonnam National University, Gwangju 61186, South Korea; Department of Artificial Intelligence Convergence, Chonnam National University, Gwangju 61186, South Korea; Department of Nuclear Medicine, Chonnam National University Hospital, Gwangju 61469, South Korea; Department of Artificial Intelligence Convergence, Chonnam National University, Gwangju 61186, South Korea

**Keywords:** Multimodal longitudinal data, modality uncertainty, variational autoencoder, recurrent gate, Alzheimer’s disease progression

## Abstract

Alzheimer’s disease (AD) is a global neurode-generative disorder that affects millions of individuals worldwide. Actual AD imaging datasets challenge the construction of reliable longitudinal models owing to imaging modality uncertainty. In addition, they are still unable to retain or obtain important information during disease progression from previous to followup time points. For example, the output values of current gates in recurrent models should be close to a specific value that indicates the model is uncertain about retaining or forgetting information. In this study, we propose a model which can extract and constrain each modality into a common representation space to capture intermodality interactions among different modalities associated with modality uncertainty to predict AD progression. In addition, we provide an auxiliary function to enhance the ability of recurrent gate robustly and effectively in controlling the flow of information over time using longitudinal data. We conducted comparative analysis on data from the Alzheimer’s Disease Neuroimaging Initiative database. Our model outperformed other methods across all evaluation metrics. Therefore, the proposed model provides a promising solution for addressing modality uncertainty challenges in multimodal longitudinal AD progression prediction.

## I. Introduction

ALZHEIMER’S disease (AD) represents a growing global health challenge with profound social and economic implications. It is the most common form of dementia, affecting millions of individuals worldwide. Recent estimates [1], [2] have shown that the number of Alzheimer’s cases has reached a staggering figure globally, with millions of new diagnoses each year. The impact of Alzheimer’s on daily life is profound, not only for those directly affected but also for their families and caregivers. However, there are treatments available only for the early-onset stages of AD, including normal cognitive (NC) and mild cognitive impairment (MCI) [3], [4]. Therefore, proactive measures in diagnosing and anticipating AD progression are essential for developing an optimal treatment plan for patients at early stages.

Recently, the integration of multimodal data in deep-learning models has become increasingly prevalent and has exhibited several advantages across various domains. In particular, the combination of neuroimaging modalities, including positron emission tomography (PET) and magnetic resonance imaging (MRI), has yielded significant performance improvement in many applications in the medical field such as diagnosis [5], [6], [7], segmentation [8], and survival analysis [9]. Therefore, most researchers [10], [11], [12], [13], [14] combine information from unimodal neuroimaging (MRI or PET) to diagnose the clinical status or predict the AD progression. Moreover, other researchers [15], [16], [17], [18] have demonstrated superior performance diagnostic outcomes when using multimodal neuroimaging MRI and PET data. However, these methods cannot solve the issue of modality uncertainty in practice, primarily because this may be due to the high cost of medical imaging. Additionally, when using longitudinal multimodal data, it is common to encounter several types of missing data, such as arbitrary missing modalities, partially missing visits, and completely missing visits. These missing data, regarded as modality uncertainty issue, can adversely affect the quality and reliability of disease prediction.

To address this data uncertainty issue, previous methods have applied various variational autoencoders [19], [20] or generative adversarial networks (GANs) [21] to learn correlations among modalities for AD diagnosis. However, these methods are not designed for disease progression, especially for AD predictions using longitudinal data. In addition, these methods cannot reconstruct the observed modality and generate the missing modality, which is essential for supporting the learning of more meaningful feature representations.

Additionally, longitudinal data are commonly processed with recurrent neural networks (RNNs), such as long short-term memory (LSTM) networks. The LSTM mechanism is specifically designed to tackle the vanishing gradient problem, enabling the capture of long-term dependencies in sequential data. Specifically, the forget gate is responsible for determining which information needs to be discarded or retained from the cell state. However, when the training data are not well-scaled, the output values of the forget gate may not be distributed well [22]. This can impact the model’s ability to learn and retain information over long sequences. Therefore, the conventional forget gate should be reformulated to enhance the ability to remember valuable information and alleviate irrelevant ones. In this study, we propose a model that can extract and constrain each tested modality into a common representation space to capture intermodality interactions among different modalities considering modality uncertainty. It is an end-to-end longitudinal multimodal disease progression prediction network (LMDP-Net) used to predict AD progression in the followup years. In addition, we introduce a novel auxiliary function to enhance the learning of temporal longitudinal representation which effectively retains optimal information flow over the time points. The contributions of this study can be summarized as follows:

- The modality uncertainty problem is solved to allow predictions of the followup years using multimodal neuroimaging, biomarkers, genetics, and demographics. Our model, known as LMDP-Net, can operate in diverse practical situations, such as those associated with current visit information only, and with past and current visit information cases associated with data ambiguity.
- We propose a module (called *Multimodal Neuroimaging Representation Fusion* (M3VAE)) to learn the fused image representation from multimodal neuroimaging data, which include MRI and PET images. This module handles the modality ambiguity problem by merging all observed image representations with the same distribution as it learns and transforms the latent representation of each modality into a common distribution.
- We propose an encoding module (called IRLSTM) to maintain and update the cell state over time by integrating an auxiliary function into the forget gate of the LSTM mechanism. This module enables the model to capture long-term dependencies effectively and robustly in longitudinal data cases.
- We conduct comprehensive experiments on the data collected from the AD Neuroimaging Initiative (ADNI) database, which is the largest AD database. The experimental results demonstrate that our proposed method outperforms the state-of-the-art methods in terms of all the evaluation metrics.
  The subsequent sections of this paper are organized as follows: Section II provides an overview of previous studies related to predictions on disease progression. The specifics of the proposed model are outlined in Section III. Section IV presents the experiment results, along with a comparison to existing methods. Section V provides the visualization, analysis, and discussion. Finally, Section VI encapsulates our findings and conclusions.

## II. Related Work

In this section, we present a thorough review of previous research endeavors focused on disease progression prediction. Sukkar et al. [23] used four input features, including the hippocampus volumes, changes in the hippocampus between successive visits, ventricular volumes, and changes in the ventricles between successive visits. Six Hidden Markov Models were employed to predict the status of AD in six subsequent visits. Doody et al. [24] estimated the correlations between different cognitive scores using the effects of linear regression models. However, these approaches did not consider the data uncertainty challenge or the use of imaging data.

Recently, several RNN-based approaches [25], [26], [27], [28], [29], [30] have been proposed to handle uncertain variables in sequential data. GRU-D model [29] employed two trainable decays, one for combining the global mean values and the last predicted values from the previous visit to impute the uncertain values while the other was integrated into the GRU model to decrease influences of historical information when these were missing. MRNN model [30] employed a bidirectional RNN, which incorporated hidden states from both forward and backward directions to impute uncertain values and predict disease status. BRIST model [27] imputed the uncertain values by considering the temporal and multivariate relations among input features. The imputed values were then fed into the encoding module of a bidirectional RNN model to predict the subsequent variables and clinical status. LSTM-T model [28] adapted the imputation module of the BRIST [27] model to impute the uncertain values and incorporated the masking vector into the encoding module of the vanilla LSTM to inform the model in which the imputed values were derived from (imputation module).

Ho et al. [26] proposed a multi-task learning (called BiPro) to address three issues including biomarker forecasting, uncertain value imputation, and prediction of disease status. They imputed the uncertain values by incorporating both statistical and dynamic features. Additionally, others [31], [32], [33] leveraged the capability of GANs in capturing the complex distribution and temporal dependencies of input features in time-series data for imputing uncertain values. However, the encoding modules of these aforementioned methods are based on variants of RNNs including GRU and LSTM. These RNN models summarize information from the current input and the previous hidden state via gated weights. Therefore, the current information is prone to lose impact as it propagates through several consecutive time points. To address this issue, we reformulated the recurrent gate (e.g., the forget gate in LSTM) to enable the model to retain more useful information from the previous time points while alleviating the irrelevant one.

Another issue is that the aforementioned approaches do not include imaging modality features as part of the input features. In contrast, the integration of imaging modalities significantly enhances the diagnostic capabilities available to medical experts. Lei et al. [34] proposed a feature selection model using extracted MRI features to predict clinical scores. Ouyang et al. [35] combined respectively convolutional neural network (CNN) and RNN mechanisms to extract latent features from MRI and predict disease status. Ho et al. [36] et al. employed biomarkers, demographics, cognitive scores, and MRI to predict disease progression using a stacked polynomial attention network. However, these approaches only considered MRI as an imaging modality and did not use PET images that can detect early biological changes in the brain associated with AD, such as the accumulation of beta-amyloid plaques and tau tangles.

Several studies incorporated both MRI and PET to diagnose the disease status. However, to the best of our knowledge, the study by Xu et al. [37] is the first study that combined multimodal imaging data to predict the disease status in followup years, despite the arbitrarily missing imaging modalities. This method stacked the imaging modality uncertainty issue by finding a latent representation that can reconstruct input modalities at each time point and each patient using degradation modules. However, we have determined that this method is not suitable for practical applications. This is because the degradation modules still need to update parameters to find a suitable latent presentation before predicting new input data after the completion of model training.

MVAE [38] and MMVAE [39] models have proposed the learning of the fused latent representation from multimodal data and reconstruction of the input data. They used a dedicated encoder for each modality and regularized the latent features of each modality into a well-known distribution before merging them. The fused latent feature inherits this well-known distribution by ensuring that all latent features follow the same distribution. Even when some modalities are missing, the distribution of the fused latent feature remains unchanged, thus enabling it to be used for reconstructing the input data. Consequently, these methods can address the modality uncertainty issue. However, they are associated with certain limitations; MVAE struggles to merge latent spaces from one or a subset of all modalities, while MMVAE excels in cross-modality generation but falls short in producing a sharp distribution in the latent space from multimodal data. In this study, we propose a model to learn effectively a fused latent representation that can address the aforementioned drawbacks. Our proposed method can produce sharper distributions of the latent space from both unimodal and arbitrary subsets of all available modalities. We employ the product-of-expert method (PoE) [40] to generate the latent spaces with a sharp distribution and then fed them into multiple decoders to reconstruct all modalities.

## III. Proposed Methods

**Fig. 1.**
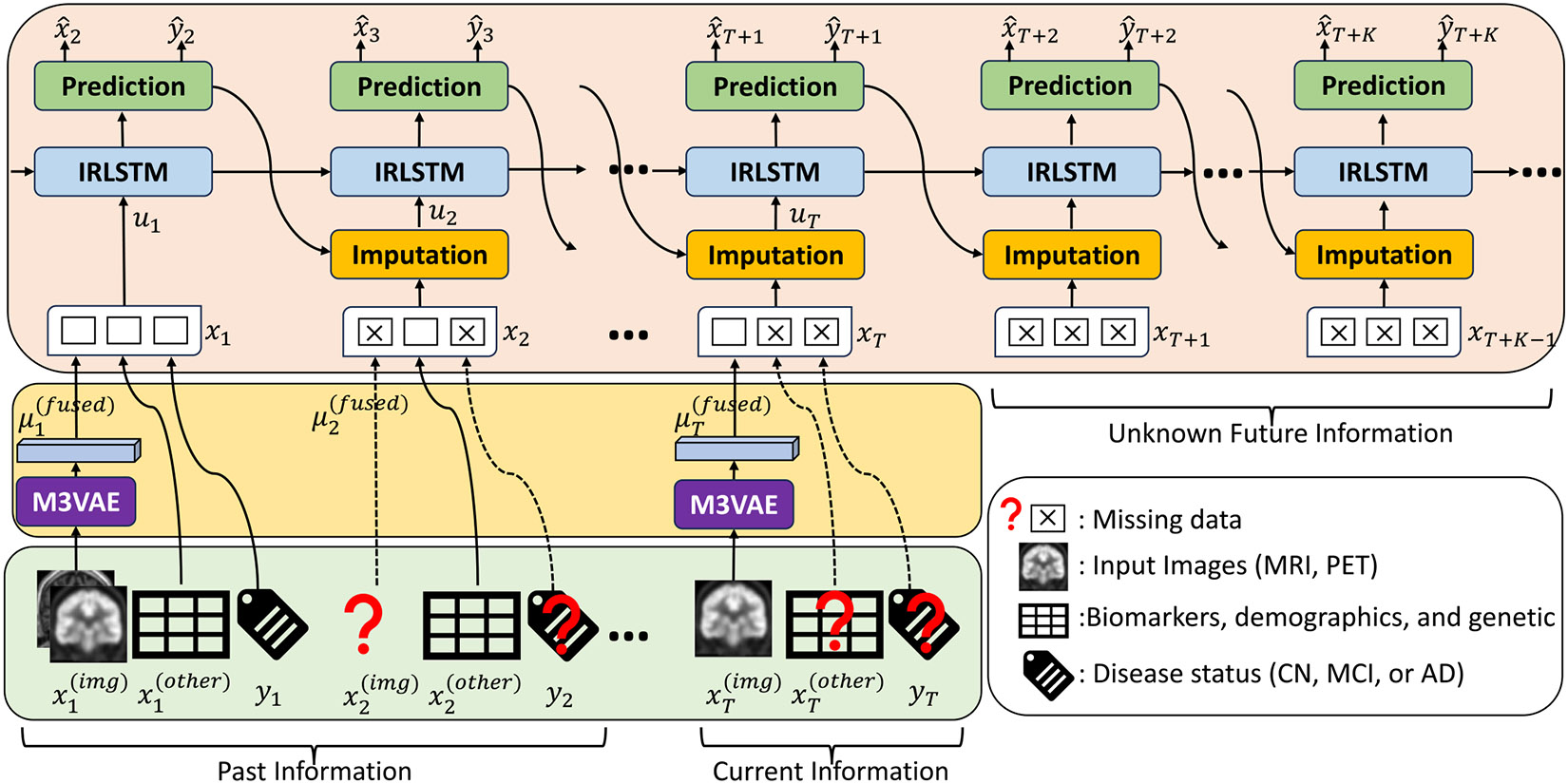
Overall architecture of longitudinal multimodal disease progression prediction network (LMDP-Net).

In this section, we describe our model to predict longitudinal AD progression (LMDP-Net) that consists of four main modules: *M3VAE*, *Imputation*, *Encoding (IRLSTM)*, and *Prediction*. M3VAE is used to extract the fused latent representation of multimodal neuroimaging to solve the issues pertaining to the absence of any specific image modalities. The *Imputation* module aims to fill in information about missing visits and followup visits. IRLSTM is used to enhance the ability to remember valuable information by reformulating the forget gate. Finally, the *Prediction* module uses the learned information from the *Encoding* module to predict the clinical status and estimate the bioinformatics of the patient during followup visits. The overall architecture of our proposed method is illustrated in Fig. 1.

### A. Notations

To enhance comprehension, we summarize the primary symbols and notations employed in this study and their descriptions, in Table I. We first hypothesize that the current visit of an individual is the $T^{th}$ visit. We denote the recorded data as $X^{mod}=\{x_{1}^{mod},x_{2}^{mod},\ldots ,x_{T+K}^{mod}\}$, where $x_{t}^{mod}$ represents the recorded data at the $t^{th}$ visit, and *mod* indicates the type of data. For each individual, we denote MRI and PET as imaging modalities $X^{img}$, while biomarkers, genetics, and demographics are denoted as non-image features $X^{other}$. Let $Y=\{y_{1},\ldots ,y_{T},y_{T+1},\ldots ,y_{T+K}\}$ represent the diagnosis set at (T+F) visits, where T-1 is the number of historical visits and F is the number of followup visits.

**Fig. 2.**
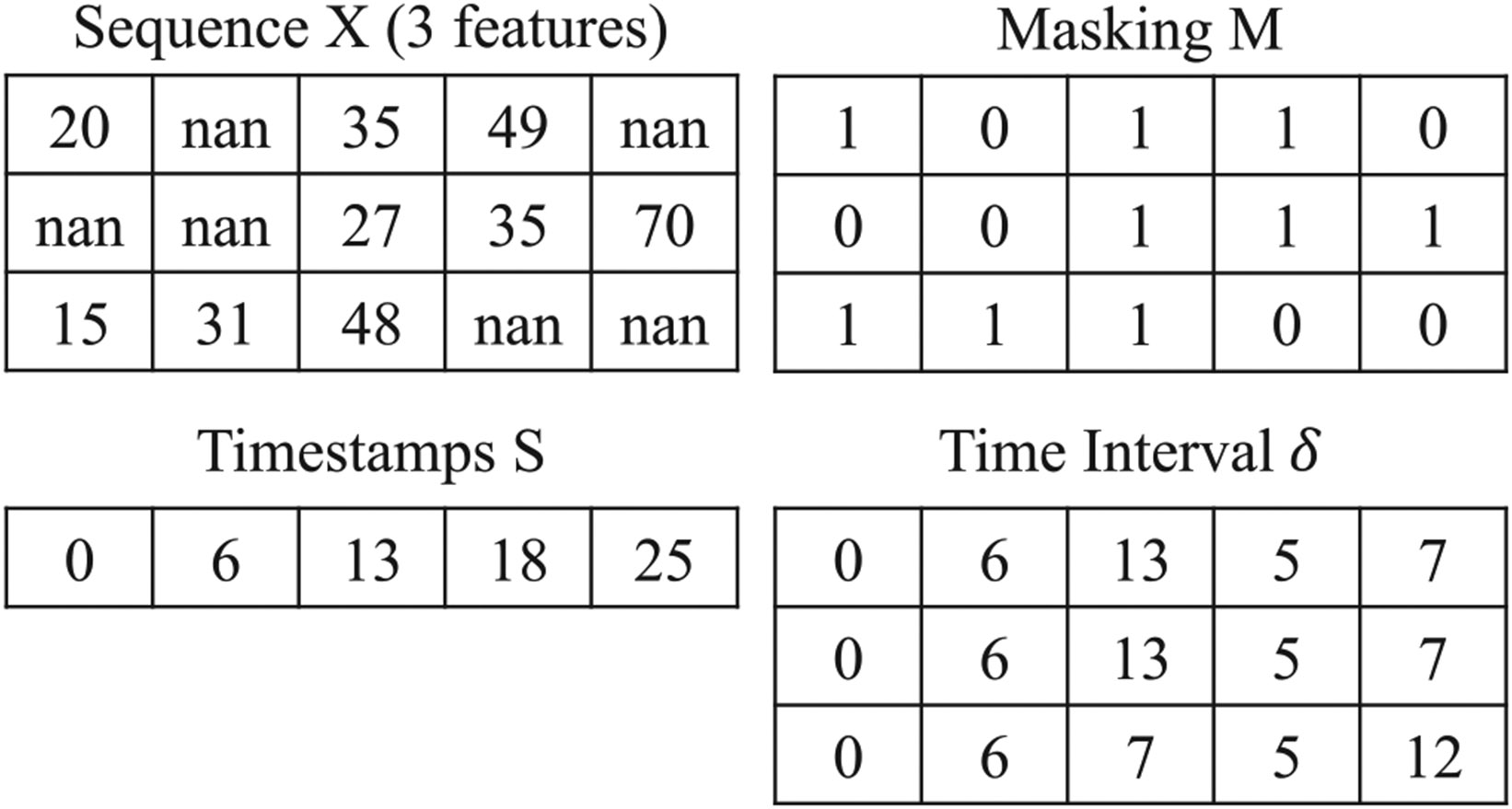
An example of multivariate sequence with uncertain values.

Let $\delta_{t}$ denote the latency from the last observed visit until the $t^{th}$ visit. We provide a masking vector $m_{t}\in \{0,1\}$ to represent which elements are observed at the $t^{th}$ visit. The variables $\delta_{t}$ and $m_{t}$ are defined as follows (see (1) and (2)),

(1) mt,emod={1ifxt,e=10otherwise}

(2) δt,e={st−st−1+δt−1,eift>1,mt−1,emod=0st−st−1else ift>1,mt−1,emod=10else ift=1}

where $m_{t,e}$, $\delta_{t,e}$, $s_{t}$, and mod are the mask, interval time, timestamp of $e^{th}$ input element at $t^{th}$, and type of input data, respectively. Fig. 2 shows an example of the notations.

### B. Modality Uncertainty

**Fig. 3.**
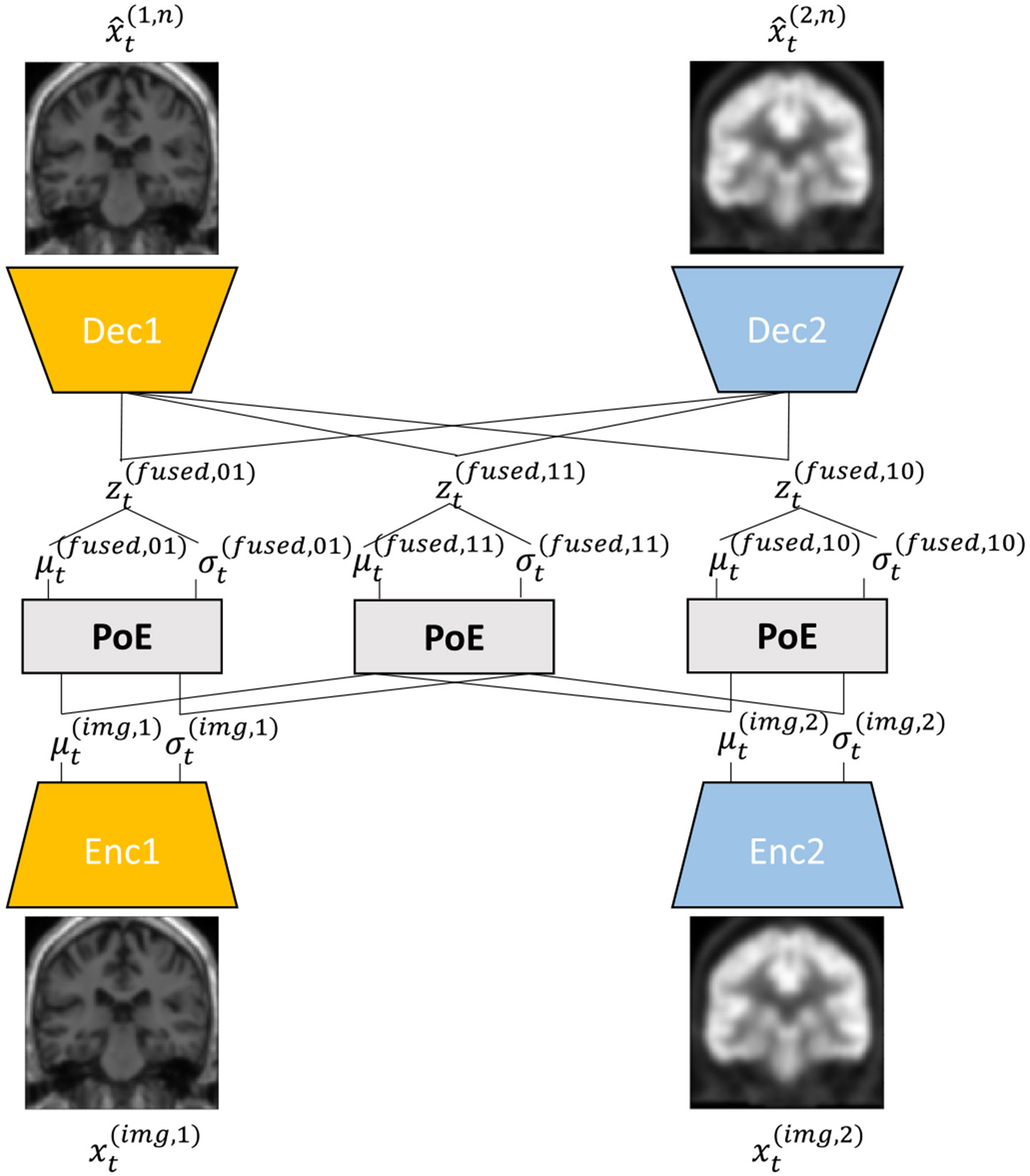
Architecture of multimodal neuroimaging representation fusion module (M3VAE) at $t^{th}$ visit.

This module aims to extract the latent representation of multimodal neuroimaging by focusing on MRI and PET, as shown in Fig. 3. We assume that if the MRI is uncertain (missing), the PET image will take on the role of the uncertain MRI. Therefore, the latent representations of the MRI and PET should be similar or approximate. To address the problem of multimodal imaging uncertainty, we first extracted latent representation from each modality using each modality-specific encoder. These were transformed into a common parameterized distribution (e.g., Gaussian distribution) that is parameterized by a resampled mean $\mu_{t}^{\left(img,c\right)}$ and resampled standard deviation $\sigma_{t}^{\left(img,c\right)}$ (see (3) and (4)).


(3) $$\mu_{t}^{\left(img,c\right)}=W_{\mu }^{\left(c\right)}(\Phi_{\left(c\right)}(x_{t}^{\left(img,c\right)}))$$

(4) $$\sigma_{t}^{\left(img,c\right)}=W_{\sigma }^{\left(c\right)}(\Phi_{\left(c\right)}((x_{t}^{\left(img,c\right)}))$$

We then adopted the PoE method [40] for merging by generating a fused latent representation (see (5), (6), and (7)). The fused latent representation is also expected to conform to a Gaussian distribution when merging all modality-specific latent representations as all these representations are assumed to adhere to a Gaussian distribution. Therefore, the fused latent representation still exhibits the properties of a Gaussian distribution even when an imaging modality is missing.

(5) $$T_{t}^{\left(img,c\right)}={\left(\sigma_{t}^{\left(img,c\right)}\right)}^{-2}$$

(6) $$\sigma_{t}^{\left(fused,n\right)}={\left(\sum T_{t}^{\left(img,c\right)}m_{t}^{\left(img,c\right)}\right)}^{-0.5}$$

(7) $$\mu_{t}^{\left(fused,n\right)}=\left(\sum \mu_{t}^{\left(img,c\right)}T^{{\left(img,c\right)}_{t}}m_{t}^{\left(img,c\right)}\right){\left(\sigma_{t}^{\left(fused,n\right)}\right)}^{2}$$

where $\mu_{t}^{\left(img,c\right)}$ and $\sigma_{t}^{\left(img,c\right)}$ are the sampled mean and standard deviation of latent features of the $c^{th}$ modality at the $t^{th}$ *visit*, respectively, $\Phi_{\left(c\right)}$ is a set of trainable parameters of an encoder for the $c^{th}$ modality, and $W_{\mu }^{\left(c\right)}$ and $W_{\sigma }^{\left(c\right)}$ are trainable parameters for the sampling mean and standard deviation from the output of encoder for the $c^{th}$ modality.

Moreover, to extract the fused latent representation in a more meaningful way, we sampled multiple fused latent representations $z_{t}^{\left(fused,n\right)}$ using $\mu_{t}^{\left(fused,n\right)}$ and $\sigma_{t}^{\left(fused,n\right)}$ (see (8)), where n denotes a binary code among $2^{C}-1$ binary codes. For example, with two imaging modalities, we will extract the fused latent representations $z_{t}^{\left(fused,01\right)}$, $z_{t}^{\left(fused,10\right)}$, and $z_{t}^{\left(fused,11\right)}$ that are only associated with the 1*^st^* imaging modality, 2*^nd^* imaging modality, and combinations of the 1*^st^* and 2*^nd^* imaging modalities, respectively. Finally, we sequentially fed each one of elements of $\left\{z_{t}^{\left(fused,01\right)};z_{t}^{\left(fused,10\right)};z_{t}^{\left(fused,11\right)}\right\}$ into the modality-specific decoders $\Theta_{\left(c\right)}$ to reconstruct the input images (see (9)). This ensures that our module can reconstruct both the observed and uncertain modalities using arbitrary combinations of the observed modalities. Therefore, we can extract and fuse all the observed imaging modalities into a fused latent representation effectively.

(8) $$z_{t}^{\left(fused,n\right)}=\mu_{t}^{\left(fused,n\right)}+\sigma_{t}^{\left(fused,n\right)}⊙𝒩(0,1)$$

(9) $${\hat{x}}_{t}^{\left(c,n\right)}=\Theta_{\left(c\right)}(z_{t}^{\left(fused,n\right)})$$

where $\Theta_{\left(c\right)}$ is the decoder of $c^{th}$ modality, 𝒩(0,1) is a Gaussian distribution, and ${\hat{x}}_{t}^{\left(c,n\right)}$ is the $c^{th}$ reconstructed modality from a set of input modalities with binary code n.

Additionally, it is important to note that the $\mu_{t}^{\left(fused\right)}$ will be used for downstream tasks instead of $z_{t}^{\left(fused,n\right)}$ because $z_{t}^{\left(fused,n\right)}$ introduces randomness 𝒩(0,1), thus leading to more diverse representations for the same input. The fused sampled mean latent representation $\mu_{t}^{\left(fused\right)}$ for the downstream task can be selected as follows (see (10)),

(10) μt(fused)={μt(fused,10)if MRI is observed,PET is missingμt(fused,01)if MRI is missing,PET is observedμt(fused,11)if MRI and PET are observed}

We concatenate the fused image feature representation $\mu_{t}^{\left(fused\right)}$ and non-image features $x_{t}^{\left(other\right)}$ (e.g., biomarkers, demographics, and genetics) as longitudinal input $x_{t}$. Additionally, we use the diagnosis $y_{t}$ as the input by concatenating with $x_{t}$. This assists our model in knowing the disease status and its progression from past visits. We use the predicted values from the previous visit and the masking vector $m_{t}$ to impute the other modalities’ uncertain values. It is important to note that we used the global mean to impute uncertain values without using this imputation module on the first visit. The way to measure imputed values is described as follows (see (11)-(15)):

(11) $$x_{t}=[\mu_{t}^{\left(fused\right)},x_{t}^{\left(other\right)}]$$

(12) $${\tilde{u}}_{t}=[x_{t},y_{t}]$$

(13) $$m_{t}=\left[m_{t}^{\left(img\right)},m_{t}^{\left(other\right)},m_{t}^{\left(y\right)}\right]$$

(14) $${\hat{u}}_{t}=[{\hat{x}}_{t-1},{\hat{y}}_{t-1}]$$

(15) $$u_{t}=m_{t}⊙{\tilde{u}}_{t}+(1-m_{t})⊙{\hat{u}}_{t}$$

where [,] denotes the concatenation operation and ⊙ denotes the element-wise multiplication.

### C. Optimization of Information Flow

**Fig. 4.**
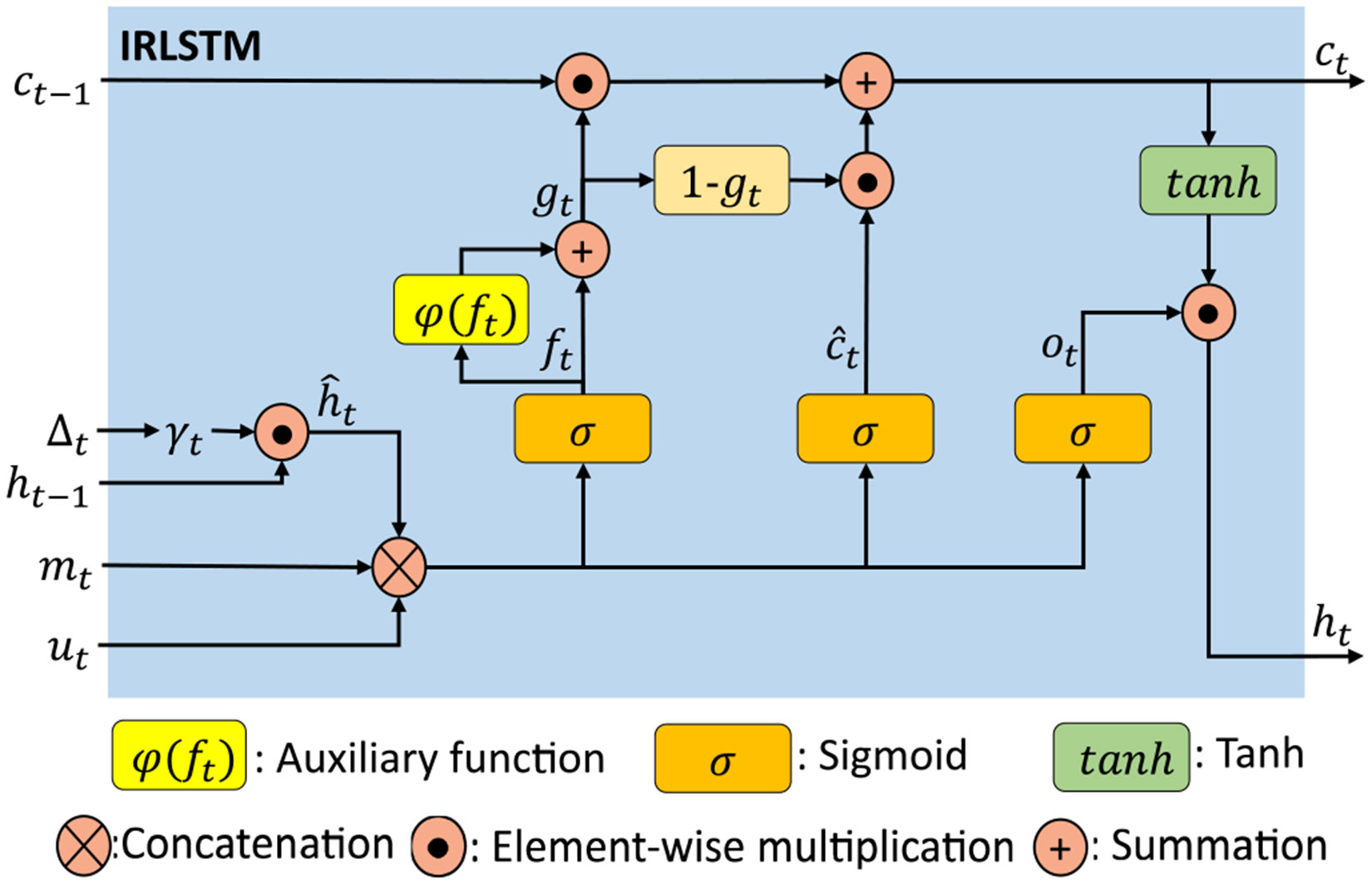
Architecture of encoding module (IRLSTM).

We modeled an auxiliary function to enhance the ability of recurrent gates robustly and effectively in controlling the flow of information over time using longitudinal data. The auxiliary function is integrated into the forget gate for temporal feature learning in longitudinal data, as shown in Fig. 4. It robustly addresses the aforementioned issue, including the capability of the forget gate to distinguish between observed and imputed data. We employed a hidden decay weight $\gamma_{h_{t}}$, derived from the interval time $\delta_{t}$, to estimate the estimated current hidden state ${\hat{h}}_{t}$. If the value of $\delta_{t}$ is high, the value of $\gamma_{h_{t}}$ will be low, and vice versa. Therefore, this property helps the model to filter knowledge from previous visits to optimize the information flow. The value of ${\hat{h}}_{t}$ is estimated as follows (see (16) and (17)):

(16) $$\gamma_{h_{t}}=e^{-(\max(0,W_{\gamma_{h}}\delta_{t}+b_{\gamma_{h}}))}$$

(17) $${\hat{h}}_{t}=h_{t-1}⊙\gamma_{h_{t}}$$

where $W_{\gamma_{h}}$ and $b_{\gamma_{h}}$ are weight and bias parameters, respectively.

Then, the masking vector $m_{t}$ is incorporated into the calculations of the gates to have information about observed or uncertain data. The mathematical expressions for the update functions of the proposed module are given by the following equations (see (18)-(20)):

(18) $${\hat{c}}_{t}=\sigma (W_{uc}u_{t}+W_{hc}{\hat{h}}_{t}+W_{mc}m_{t}+b_{c})$$

(19) $$o_{t}=\sigma (W_{uo}u_{t}+W_{ho}{\hat{h}}_{t}+W_{mo}m_{t}+b_{o})$$

(20) $$f_{t}=\sigma (W_{uf}u_{t}+W_{hf}{\hat{h}}_{t}+W_{mf}m_{t}+b_{f})$$

Typically, the updated cell state $c_{t}$ is formulated as follows (see (21)):

(21) $$c_{t}=f_{t}⊙c_{t-1}+(1-f_{t})⊙{\hat{c}}_{t}$$

However, as mentioned, the output values of the forget gate should be closer to zero or one to ensure improved discarding or retaining of information from the previous state. Therefore, we provide a formula that combines the forget gate and an auxiliary function $\psi (f_{t})$ to calculate the new forget gate $g_{t}=f_{t}+\psi (f_{t})$. The new forget gate $g_{t}$ should have similar properties as those of the Sigmoid function [22], [25]:

1. Differentiability: It is differentiable everywhere, which is crucial for gradient-based optimization algorithms. Differentiability enables the use of backpropagation for training neural networks.
2. Boundedness: The new forget gate $g_{t}$ maps its input to the range [0, 1] to control the flow of information.
3. Monotonicity: The $g_{t}$ value must be monotonically increasing in the range [0, 1].
4. Symmetricity: It should be symmetric around 0.5in terms of geometric sense that may be preferred for creating balanced and harmonious patterns.

**Fig. 5.**
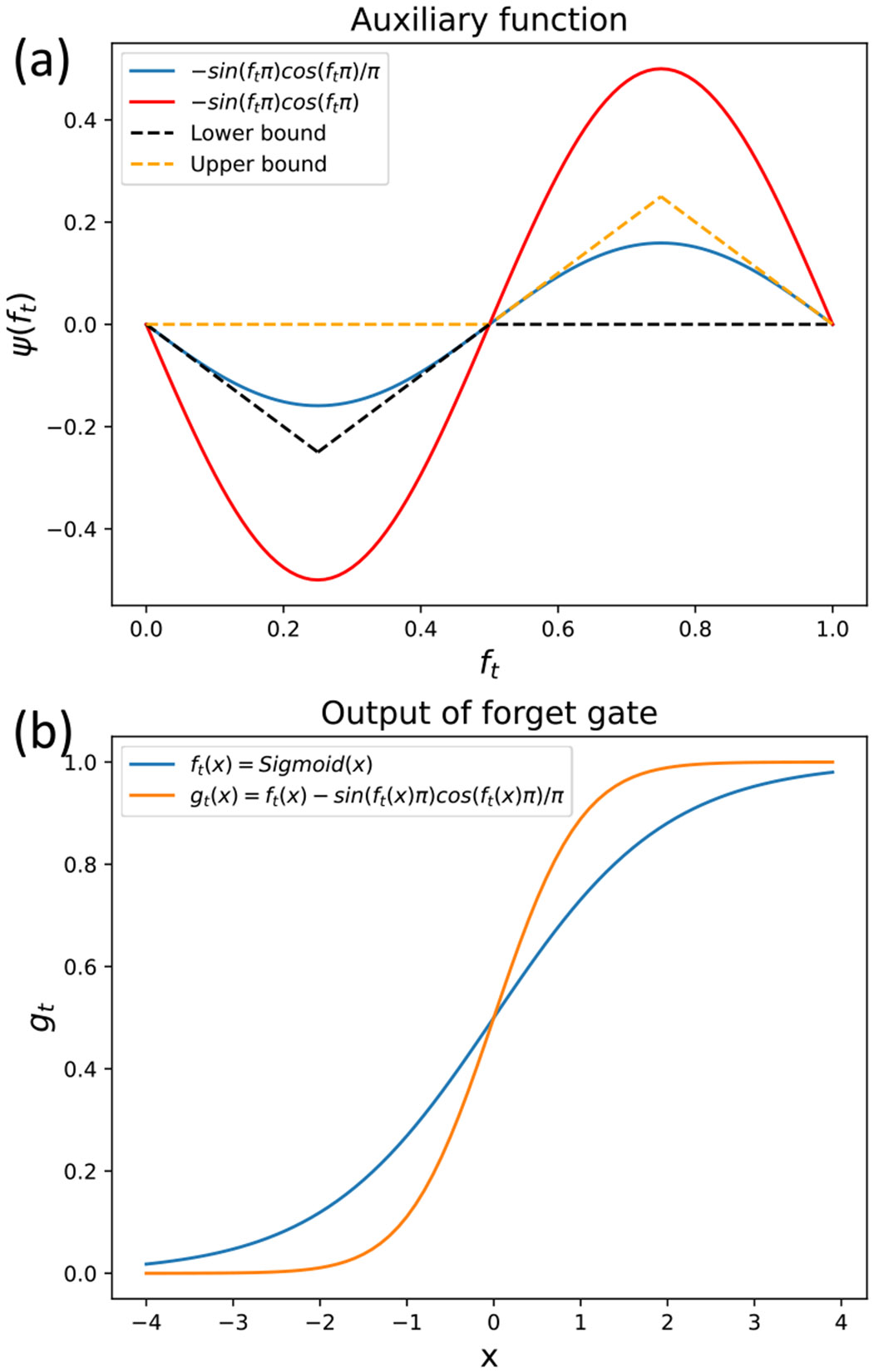
(a) Auxiliary function. Note that the value of auxiliary function should be greater than lower bound and smaller than upper bound. (b) Forget-gate outputs before and after incorporating the auxiliary function.

To ensure that the new forget gate $g_{t}$ value is closer to zero or one, the auxiliary function $\psi (f_{t})$ should be positive when $f_{t}>0.5$; otherwise, $\psi (f_{t})$ should be negative when $f_{t}<0.5$. We observed that $\psi (f_{t})=-\sin(f_{t}\pi )\;\cos(f_{t}\pi )$ perfectly satisfies the above condition. It also meets the *Differentiability* property. However, it is important to note that the output values of $f_{t}$ and $g_{t}$ are between zero and one. Therefore, the absolute value of $\psi (f_{t})$ must be lower than $\text{min}(f_{t},1-f_{t})$, or equivalently, $\psi (f_{t})\in [-0.5,0.5]$, to satisfy the *Boundedness* property. Unfortunately, the value of the function $-sin(f_{t}\pi )\;\cos(f_{t}\pi )$ is not always in the range [−0.5, 0.5], as shown in Fig. 5(a). Hence, we need to identify a factor β to reduce the value of $-sin(f_{t}\pi )\;\cos(f_{t}\pi )$ by dividing it by β. We assumed that $g_{t}=f_{t}-\frac{sin(f_{t}\pi )\;\cos(f_{t}\pi )}{\beta }$ satisfies the *Boundedness* property. The function $g_{t}$ is required to be monotonically increasing; therefore, the derivative of $g_{t}$ is always non-negative over an interval (meaning that $g_{t}^{\prime }=1-\frac{\pi \;\cos(2\pi f_{t})}{\beta }>=0$ over $f_{t}\in [0,1]$). Therefore, β must be greater than or equal to π to satisfy both *Boundedness* and *Monotonicity* properties. We set β=π so that the value of $g_{t}$ is as close to zero or one as possible. Consequently, the new forget gate $g_{t}$, shown in Fig. 5, is formed as follows (see (22)):

(22) $$g_{t}=f_{t}-\frac{\sin(f_{t}\pi )\;\cos(f_{t}\pi )}{\pi }$$

The current cell $c_{t}$ and hidden state $h_{t}$ are calculated as follows (see (23) and (24)):

(23) $$c_{t}=g_{t}⊙c_{t-1}+(1-g_{t})⊙{\hat{c}}_{t}$$

(24) $$h_{t}=o_{t}⊙tanh(c_{t})$$

where the hidden state $h_{t}$ serves as a summary of the information processed by the IRLSTM up to time step t. It is used as input to the next time step t+1. In addition, the prediction module uses the hidden state $h_{t}$ as input to forecast the variables of the subsequent visits including the estimated diagnosis ${\hat{y}}_{t+1}$ and the estimated multimodal data ${\hat{x}}_{t+1}$. The forecasting data generated by these predictions can serve as input for the subsequent visit, particularly in cases in which there are uncertain data. The prediction module is defined as follows (see (25) and (26)),

(25) $${\hat{x}}_{t+1}=W_{g}h_{t}+u_{t}$$

(26) $${\hat{y}}_{t+1}=Softmax(W_{y}h_{t}+b_{y})$$

Our proposed method consists of multiple tasks such as the prediction of progression, imputation, and imaging representation learning. Therefore, the overall objective function is the combination of multiple loss functions, described as follows (see (27)-(30)):

(27) $$L_{total}=L_{p}+L_{i}+L_{f}$$

(28) $$L_{p}=-\sum_{t=2}^{T+K}(y_{t}\;\log\;{\hat{y}}_{t})$$

(29) Li=∑t=2T+K∣x^t(Bio)−xt(Bio)∣Lf=−∑t=1T+K∑n=12C−1EqΦlnpΘ(xt(img,n)∣zt(fused,n))−∑t=1T+K∑n=12C−1(KL[qΦ(zt(fused,n)∣xt(img,n))∥pΘ(zt(fused,n))])

with

$$E_{q_{\Phi }}\;\text{ln}\;p_{\Theta }\left(x_{t}^{\left(img,n\right)}|z_{t}^{\left(fused,n\right)}\right)\;\;=E_{q_{\Phi }}\;\text{ln}\;\left[p_{\Theta }\left(x_{t}^{\left(img,1\right)}|z_{t}^{\left(fused,n\right)}\right)p_{\Theta }\left(x_{t}^{\left(img,2\right)}|z_{t}^{\left(fused,n\right)}\right)\right]\;\;=-MSE\left(x_{t}^{\left(img,1\right)};{\hat{x}}_{t}^{\left(1,n\right)}\right)-MSE\left(x_{t}^{\left(img,2\right)};{\hat{x}}_{t}^{\left(2,n\right)}\right)$$

and

(30) $$KL\left[q_{\Phi }\left(z_{t}^{\left(fused,n\right)}|x_{t}^{\left(img,n\right)}\right)∥p_{\Theta }\left(z_{t}^{\left(fused,n\right)}\right)\right]\;\;\approx KL\left[𝒩\left(\mu_{t}^{\left(fused,n\right)},\sigma_{t}^{\left(fused,n\right)}\right)∥𝒩(0,I)\right]\;\;=\frac{1}{2}\left({\left(\mu_{t}^{\left(fused,n\right)}\right)}^{2}+\sigma_{t}^{\left(fused,n\right)}-\text{ln}\;\sigma_{t}^{\left(fused,n\right)}-1\right)$$

where $L_{p}$, $L_{i}$, and $L_{f}$ are the disease progression prediction, imputation, and multimodal feature representation learning losses, respectively; MSE and KL are the mean-square error and Kullback-Leibler divergence losses, respectively, and $q_{\Phi }(.)$ and $p_{\Theta }(.)$ are the encoder and decoder modules, respectively.

## IV. Experimental Results and Analysis

### A. Materials and Settings

In our study, we employed the ADNIMERGE dataset (obtained from the Alzheimer’s Disease Neuroimaging Initiative (ADNI) database^1^) encompassing data from four public datasets, namely ADNI-G0, ADNI-1, ADNI-2, and ADNI-3. The ADNIMERGE dataset includes various types of data, such as demographic, exam and assessment, genetic, biomarker, and imaging obtained from 2430 participants across different sites from more than 16,340 visits. Our experiment involved demographics, biomarkers, genetic, and imaging (MRI and PET) data. Demographic information included age, gender, and education. Imaging data involved the use of both PET and MRI. Biomarkers consisted of six measurable indicators extracted from longitudinal T1-weighted MRI, providing detailed volumes of brain regions, such as Ventricles, Hippocampus, WholeBrain, Entorhinal, Fusiform, and MidTemp. By combining information from both MRI and biomarkers, we can leverage the complementary strengths of each type of data. The APOE4 biomarker was chosen as a genetic biomarker because it is associated with an increased risk of developing AD [41].

We initially limited the prediction of annual progression from baseline visits up to 5 (followup) years (i.e., M0, M12, M24, M36, M48, and M60) based on [42]. The selected participants had a minimum of two available visits along with the clinical diagnosis (including the baseline visit). We excluded participants with reversible diagnoses, specifically those who transitioned from mild cognitive impairment (MCI) to cognitive normal (CN) or from AD to either MCI or CN. Consequently, we acquired data for a total of 1369 patients with 5768 observed visits, comprising 971 stable patients and 398 progressive patients. The details of the cleaned dataset are outlined in Table II.

- Demographic data (Dg): The database only provides the age of participants at the baseline visit. Therefore, we gradually increased the age based on interval time between the baseline visit and subsequent visit. In addition, education and age are numerical variables, we standardized them by calculating the mean and standard deviation (also called z-score normalization). The gender was converted to a one-hot encoding vector.
- Genetic data (Gen): The APOE4 is encoded to a one-hot encoding vector.
- Biomarker data (Bio): To deal with variability in brain size among different patients, we first normalized six biomarkers by dividing them by individual intracranial volumes. We applied the z-score normalization.
- Imaging data: Based on [21], we employed image registration, cropping, and normalization as the primary preprocessing steps. Initially, the MRI and PET were registered to the MNI152 [43], [44] standard brain template using SPM12^2^ in MATLAB R2021a. The size of the registered image was relatively large (matrix: 182 × 218 × 182 voxels). As a result, the boundary regions of the image were discarded to reduce the size and eliminate irrelevant information related to the brain structure. The size of the cropped MRI and PET were 128 × 160 × 128. Lastly, we scaled the voxel values to a range (values between zero and one) using min-max normalization.

We employed various evaluation metrics to demonstrate the effectiveness of our proposed model. For uncertain modality reconstruction, we used mean-square error (MSE) and peak signal-to-noise ratio (PSNR). For biomarker imputation, we used meanabsolute error (MAE) and mean relative error (MRE). For the prediction of AD progression, we used four common metrics, namely accuracy (Acc), precision (Pre), recall (Rec), and mean area under the receiver operating characteristic curve (mAUC).

We conducted comprehensive experiments using the Intel Xeon Gold 6326 central processing unit and Nvidia RTX A6000 graphics processing unit to train and test our proposed model and other existing models. The Adam optimizer [45] with a learning rate of 0.002 was employed to update all the model parameters. When training, we initialize the hidden state $h_{0}$ with zeros. We randomly masked the diagnosis of the current visit and gathered all the information from the ${\left(T+1\right)}^{th}$ visit onward (t can be {T+1,T+2,…,T+K}). This allows our proposed model to use both historical and current information to predict progression in K followup visits. We included all the patients diagnosed with AD during their first visit in the training set because our goal was to predict disease progression in patients at early stages of AD. We applied a 5-fold cross-validation technique for training and testing the performance. Importantly, we used the same settings mentioned above for all competing models for fair comparisons.

### B. Uncertainty Performance

**Fig. 6.**
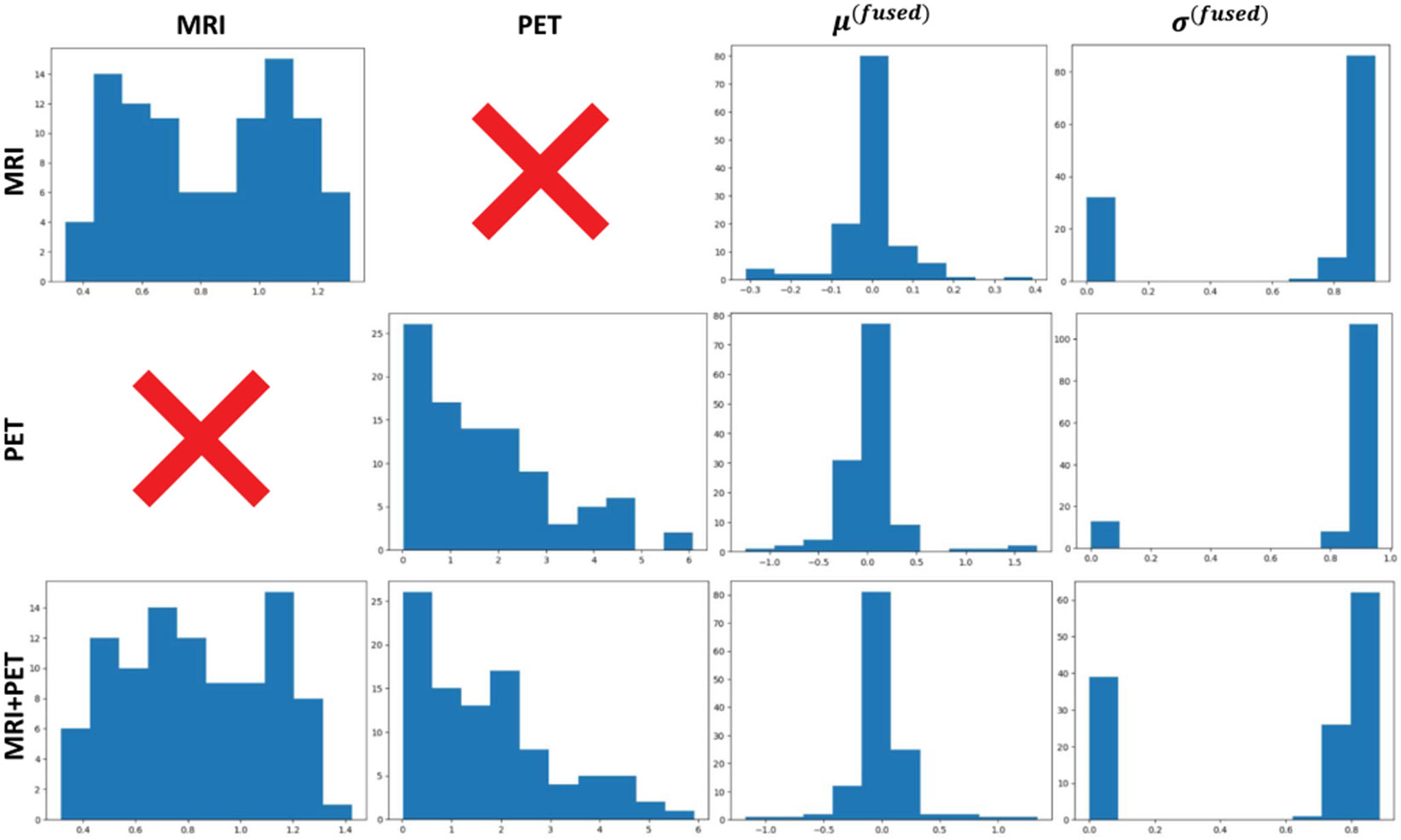
Visualization of the distribution before and after applying PoE method. Rows denote the input modalities, and columns denote the distribution of latent spaces.

We initially assessed the performance of reconstructing uncertain modalities by estimating MSE and PSNR. Our experiments were conducted under two conditions: reconstructing modalities (i) from MRI only and (ii) from PET only. We compared the proposed method with other multimodal fusion techniques, including concatenation, summation (MMVAE), and multiplication (MVAE), which are used to fuse multimodal data in latent spaces. The Table III shows that our method has better results compared with the competing methods across all scenarios. Besides, to understand the process of our multimodal fusion method, we visualize the distribution of the latent space before and after applying the PoE method, as illustrated in Fig. 6.As can be seen in the figure, before applying PoE, there are multiple distributions, each representing a different expert’s opinion. These distributions can be broad, with each expert providing a different perspective or partial information about the data. However, after applying PoE, the individual distributions are multiplied together to form a single, combined distribution. The mean $\mu^{\left(fused\right)}$ and standard deviation $\sigma^{\left(fused\right)}$ of the fused latent space are mostly close to 0 and 1, respectively.

We also conducted a comparison against existing methods by focusing on the two metrics of MAE and MRE to evaluate the performance of our proposed method in biomarker imputation tasks. The results in Table IV demonstrate that our proposed method has the lowest values for different input combinations. This indicates that the imputed values generated by our model are closer to the actual values than those produced by the existing methods. It is noteworthy that our imputation module was inspired by MinimalRNN; however, our model significantly outperforms the MinimalRNN model in terms of the MAE and MRE metrics. This observation indicates that our encoding module excels in learning and capturing essential information, surpassing the capabilities of the MinimalRNN’s counterpart.

### C. Effect of Auxiliary Function on Information Flow

**Fig. 7.**
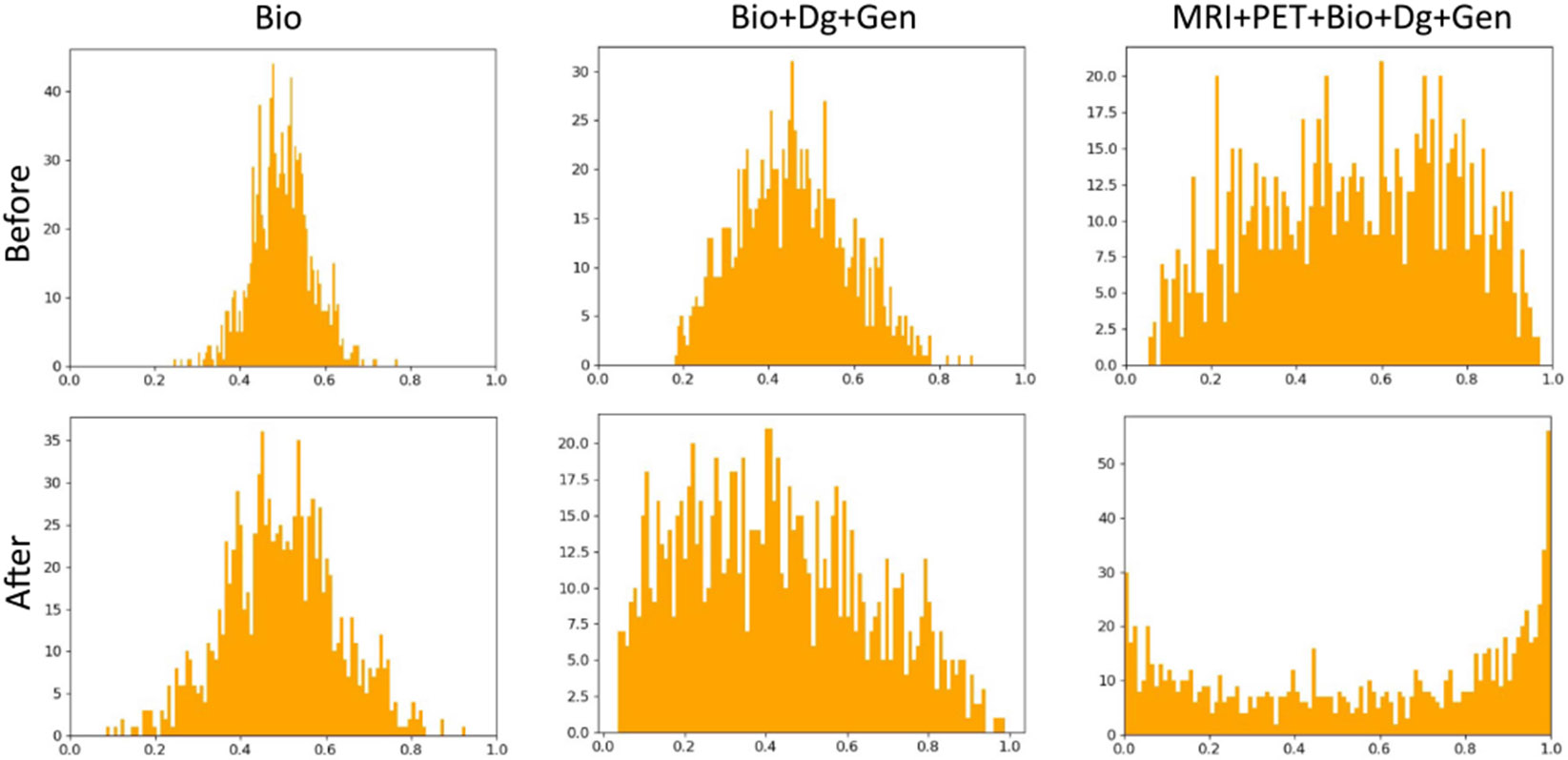
Distribution of output values of the forget gate before and after the integration of the auxiliary function.

We investigate herein the extent to which the masking vector and the auxiliary function can enhance the performance of longitudinal data prediction. Accordingly, we decomposed each component separately and performed training using all possible combinations. Table V demonstrates that integrating the masking vector can help the model identify the nonmissing input data. Furthermore, our proposed auxiliary function, combined with the masking vector, has shown superior performance compared with the vanilla LSTM model across various input data combinations, as indicated by all the evaluation metrics. Additionally, we visualize the distribution of the output values of the forget gate with and without the auxiliary function, as shown in Fig. 7. Fig. 7 shows that the output values of the forget gate are redistributed and stretched closer to zero and one after integrating our novel auxiliary function. This makes the model more effective in retaining important and discarding irrelevant information. From the results reported in Table V and Fig. 7, we can conclude that our novel auxiliary can enhance the capacity of the LSTM model robustly.

### D. Comparison of Disease Progression Predictions

When evaluating the effectiveness of our proposed method in predicting disease progression, we conducted a comprehensive comparison with existing approaches. We employed the accuracy, precision, recall, and mAUC metrics as four key performance indicators. We also conducted experiments using different input data. As shown in Table VI, our LMDP-Net method achieves the outcomes of 0.6222 ± 0.0128, 0.6338 ± 0.0138, 0.6295 ± 0.0160, and 0.7899 ± 0.0130 in terms of accuracy, precision, recall, and mAUC values when using biomarker, demographic, genetics, and multimodal imaging data. These values are significantly higher than those achieved by existing methods. Our method also outperforms competing methods across all evaluation metrics for various input combinations. Moreover, Table VI reveals that the combination of multimodal neuroimaging data, biomarkers, genetics, and demographics significantly enhances the performance of predicting disease progression compared with the performance when only non-imaging data are used.

Additionally, we also employed t-tests to assess the proposed model quantitatively. As depicted in Table VI, our proposed method exhibits significant differences compared with all competing methods and metrics, concerning accuracy, recall, and mAUC (except for the MVAE model).

## V. Visualization, Analysis, and Discussion

In this section, we visualize the predicted biomarkers and the disease progression over the five follow-up years. Additionally, we discuss our findings regarding the use of multimodal data, the incorporation of past-visit information, and the inclusion of current diagnoses as part of the input data.

### A. Effects Following the Incorporation of Past-Visit Information

We explored the effects of information from the past visits by conducting experiments by increasing the number of past visits. As shown in Table VII, the prediction performance robustly improved across all evaluation metrics. It is important to note that using only data from the current visit results in lower performance, as we assume that the clinical diagnosis from the current visit was not available. However, by incorporating information from the past visits and the current visit, the model can inherit the clinical diagnosis from the previous method. Consequently, performance is enhanced by utilizing data from both the previous and current visits. This reveals that our proposed method can be applied in practice in many situations, including the patients’ first, second, third, and subsequent visits.

### B. Effects of Using Diagnosis At the Current Visit

To investigate the effect of diagnosis, we conducted experiments with and without using it at the current visit. As shown in Table VIII, the use of a diagnosis at the current visit yields significant performance enhancements compared with the scenario in which it was not used, thus improving accuracy and precision by more than 20%, and recall and mAUC by 14%. Therefore, we can use the actual diagnosis from the current visit at the inference stage. However, the diagnosis was not used in the current visit when the model was trained because the majority of training samples had a stable disease status (not progressing). If we use the diagnosis from the current visit, the model may exhibit a bias by predominantly learning from the diagnosis information, without taking into account other information such as biomarkers, genetics, demographics, and multimodal imaging data.

### C. Comparison Between Unimodal and Multimodal Neuroimaging Data in AD Progression Predictions

We compared the performances of the cases in which using unimodal and multimodal imaging data were used to explore the effects of multimodal neuroimaging data. The listings in Table IX show that the use of multimodal data significantly enhanced performance compared with that of unimodal data across all evaluation metrics. Moreover, we observed that the use of PET images yielded better performances compared with those associated of MRI. These observations may help medical experts and patients choose a good option to predict AD progression.

### D. Experiments on Different Hidden State Sizes

This experiment examines the performance of the proposed model with different hidden state sizes. We selected hidden state sizes of 64, 128, and 256, as shown in Table X. As shown in Table X, the hidden state size of 128 achieved the best performance. The other settings, with hidden state sizes of 64 and 256, exhibited lower accuracy and higher variance in their results. However, their performances were still superior to those of other existing methods (refer to Table VI).

### E. Visualization of the Predicted Biomarkers

**Fig. 8.**
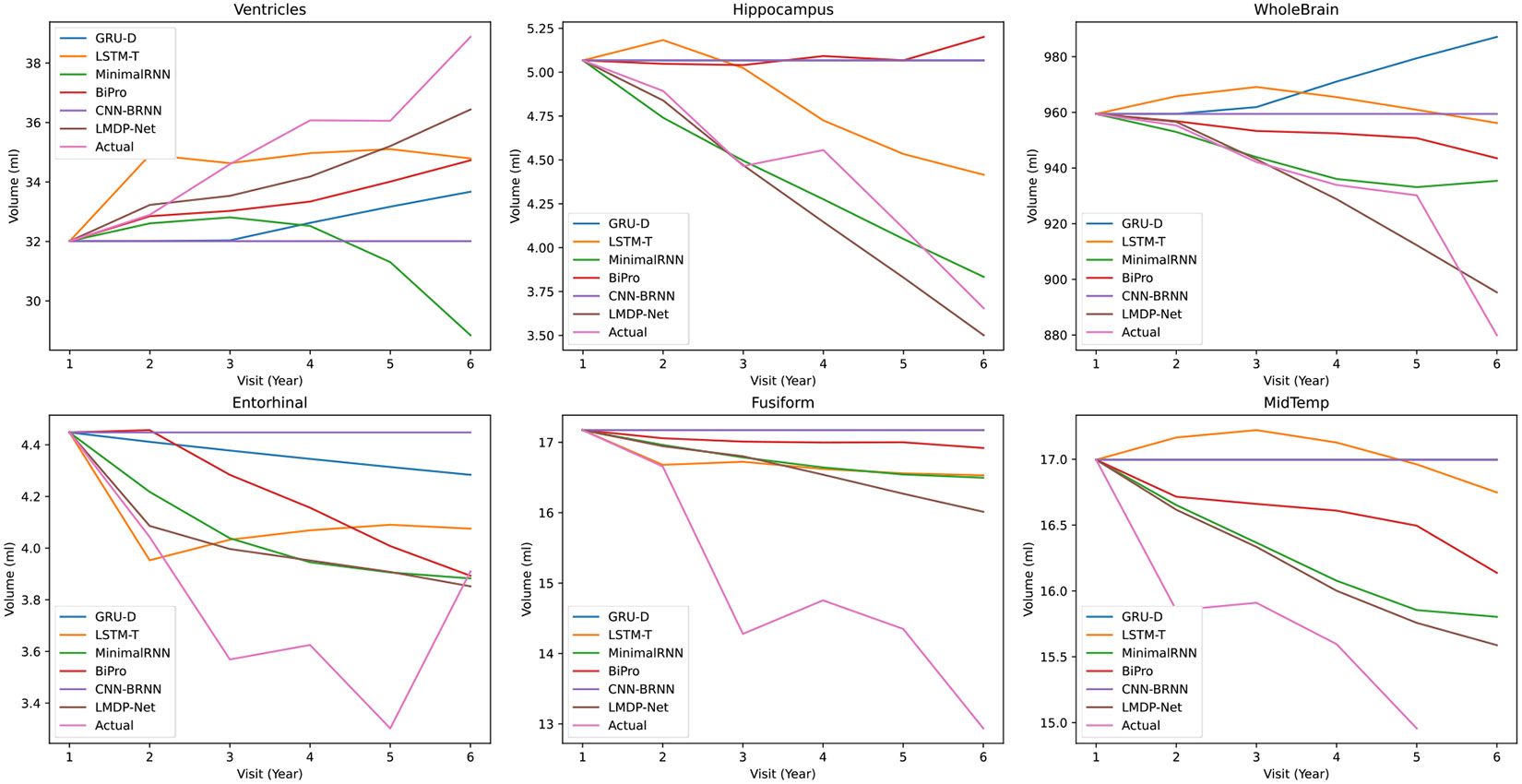
Visualization of predicted biomarkers in five followup visits after the first visit.

We visualized the predicted biomarkers in five followup visits after the first visit. As shown in Fig. 8, the predicted biomarker values based on our LMDP-Net model agreed closely with the actual values, compared with those obtained based on the competing methods. Furthermore, although there may be noise causing fluctuations in the actual values (e.g., in biomarkers such as Entorhinal, Fusiform, Midtemp, and Hippocampus) that affect the training process, the trajectory of our predicted values remains smoother than those of other methods. Additionally, we observed a consistent trend in which the volumes of all biomarkers tended to decrease over time, except the ventricle, which increased over time. Notably, our method accurately predicts this trend without any deviation, thus demonstrating its robust performance in capturing the longitudinal changes in biomarker volumes over time.

### F. Visualization of the Disease Progression in the Followup Years

**Fig. 9.**
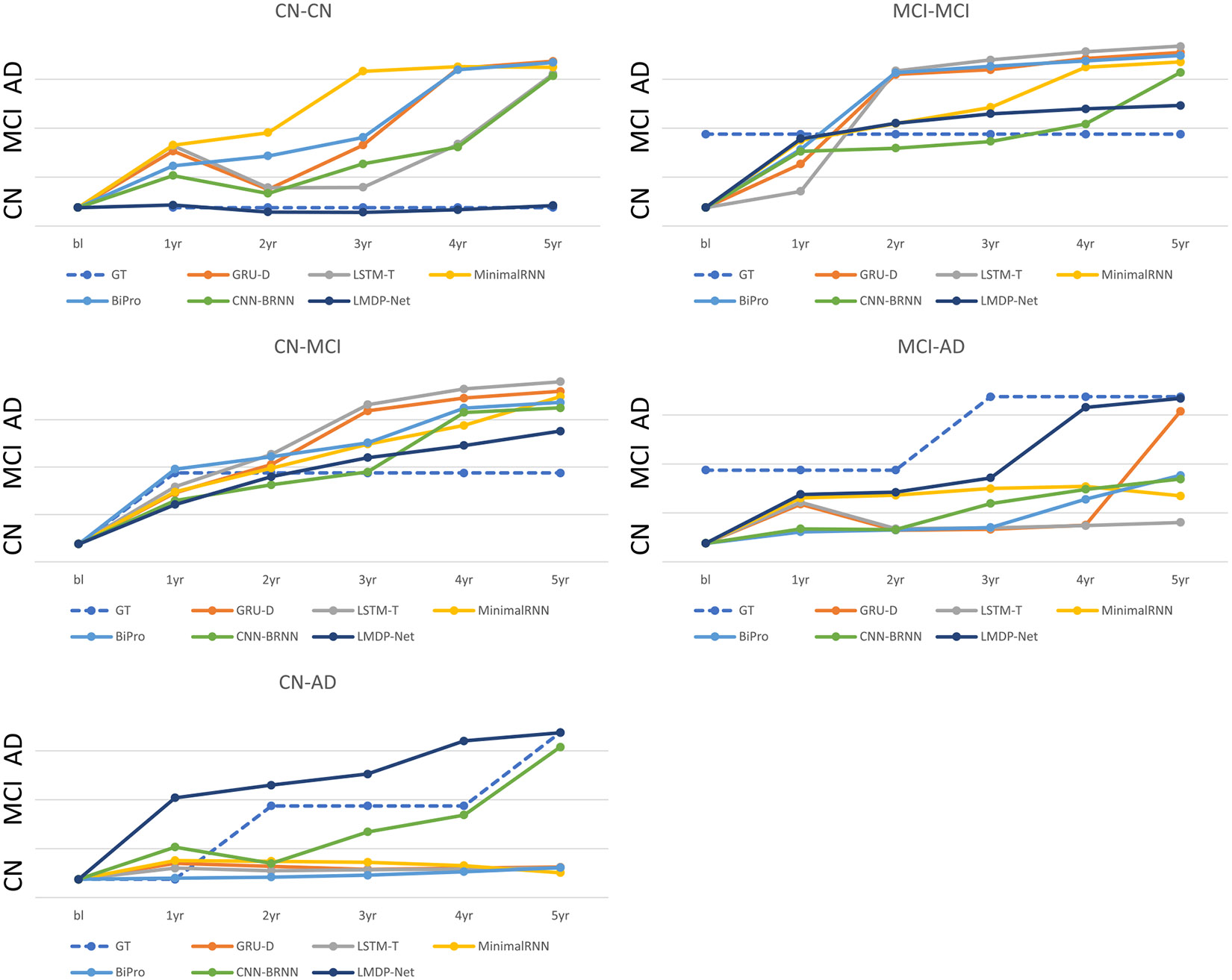
Visualization of class probability for predicted diagnoses in five followup visits following the first visit. There are five cases: stable CN (CN-CN), stable MCI (MCI-MCI), transition from CN to MCI (CN-MCI), transition from MCI to AD (MCI-AD), and transition from CN to AD (CN-AD).

We visualized the predicted progression of disease status during five followup visits following the first visit, as shown in Fig. 9. We observed that our proposed model predicts the progression more accurately compared with other methods. The predicted trajectories of the proposed method are more consistent and closer to the actual progression than those of competing methods. Furthermore, the predicted disease progression from our proposed model do not reverse from MCI to CN or from AD to MCI and CN, as observed in the GRU-D(CN-CN and MCI-AD cases), LSTM-T(CN-CN and MCI-AD cases), and CNN-BRNN(CN-CN and CN-AD cases) models. This ensures that our proposed model demonstrates a higher level of stability and reliability in longitudinal predictions.

## VI. Conclusion

In this study, we proposed a model with modality uncertainty and optimal information flow to predict disease progression in followup years using non-imaging and multimodal neuroimaging data. Our model effectively addressed the challenge of arbitrary missing modalities and introduced an auxiliary function to enhance the forget gate’s capacity in a conventional LSTM model. We observed that the combination of multimodal imaging and non-imaging data could result in improving the prediction performance. The comprehensive results showed that our proposed model outperformed existing models across all evaluation metrics. Finally, the experimental results demonstrated that our method can be applied in real-world scenarios without being adversely affected by any uncertain data.

However, our proposed method employs the PoE-based fusion method, which is computationally expensive because it requires training multiple independent encoder-decoder networks simultaneously. Additionally, the model is challenging to train due to potential issues of imbalance among modalities, leading to overfitting on the dominant modalities. Finally, our current method sets an equal weight for each task, and auxiliary tasks had the same importance as the main task. Consequently, emphasizing the main task among the auxiliary tasks has potential for us to achieve higher performance and more focused optimization, thereby improving overall model robustness and efficiency. Therefore, our future work will address the issue by adjusting dynamically the weights during the training process.

## Figures and Tables

**TABLE I:** Table of Notations

Symbol	Description
T-l	Number of previous visits until current visit
K	Number of followup visits from current visit
$y_{t}$	Diagnosis at the $t^{th}$ visit
$x_{t}^{\left(other\right)}$	Input non-imaging data (biomarker, demographic, and genetic) at $t^{th}$ visit
$x_{t}^{\left(img,c\right)}$	Input $c^{th}$ imaging modality at $t^{th}$ visit
$m_{t}^{mod}$	Masking vector of input data at $t^{th}$ visit with *mod* denotes imaging or non-imaging data
$\delta_{t}$	Time interval from last observed data until $t^{th}$ visit
$\mu_{t}^{\left(img,c\right)}$	Sampled mean latent representation of $c^{th}$ imaging modality at $t^{th}$ visit
$\sigma_{t}^{\left(img,c\right)}$	Sampled standard deviation of latent representation of $c^{th}$ imaging modality at $t^{th}$ visit
$z_{t}^{\left(fused,n\right)}$	Fused latent representation of $n^{th}$ imaging combination at $t^{th}$ visit
$\mu_{t}^{\left(fused,n\right)}$	Fused mean latent representation of $n^{th}$ imaging combination at $t^{th}$ visit
$\sigma_{t}^{\left(fused,n\right)}$	Fused standard deviation of latent representation of $n^{th}$ imaging combination at $t^{th}$ visit
${\hat{x}}_{t}^{\left(c,n\right)}$	Reconstructed image of $c^{th}$ imaging modality from the $\mu_{t}^{\left(fused,n\right)}$ at $t^{th}$ visit
n	The binary code which denotes combinations of input modalities (e.g., 01, 10, or 11 where 0 is missing and 1 is observed)
$x_{t}$	Input data (combination of imaging and non-imaging data) at $t^{th}$ visit
${\tilde{u}}_{t}$	Combination of input data and diagnosis at $t^{th}$ visit
${\hat{u}}_{t}$	Estimated imputed data at $t^{th}$ visit
$u_{t}$	Imputed input data at $t^{th}$ visit
$\gamma_{h_{t}}$	Decay weight of hidden state at $t^{th}$ visit
$h_{t}$	Hidden state at $t^{th}$ visit
${\hat{h}}_{t}$	Estimated hidden state at $t^{th}$ visit
$o_{t}$	Output gate of IRLSTM model at $t^{th}$ visit
$f_{t}$	Forget gate of IRLSTM model at $t^{th}$ visit
${\hat{c}}_{t}$	Estimated cell state of IRLSTM model at $t^{th}$ visit
$c_{t}$	Cell state of IRLSTM model at $t^{th}$ visit
$g_{t}$	New forget gate of IRLSTM model at $t^{th}$ visit

**TABLE II:** Data Statistics

	#		# (missingrate%)
Patients	1369	#MRI	4452 (45.8)
Visits	5768	#PET	1826 (77.8)
stable CN	426	#Diagnosis	5667 (31.0)
stable MCI	361	#Ventricles	4313 (47.5)
AD	184	#Hippocampus	4013 (51.1)
from CN to MCI	101	#WholeBrain	4455 (45.8)
from MCI to AD	289	#Entorhinal	3745 (54.4)
from CN to AD	8	#Fusiform	3745 (54.4)
		#MidTemp	3745 (54.4)

**TABLE III:** Uncertain Imaging Modality Reconstruction Comparison

Uncertain modality	Method	PSNR ↑	MSE ↓
PET	Concatenation	18.4843 ± 0.1123	0.0144 ± 0.0003
	MVAE [38]	18.9613 ± 0.1133	0.0131 ± 0.0003
	MMVAE [39]	18.7317 ± 0.3254	0.0139 ± 0.0010
	M3VAE	**19.0194 ± 0.1488**	**0.0130 ± 0.0005**
MRI	Concatenation	18.8408 ± 0.5140	0.0135 ± 0.0017
	MVAE [38]	19.0561 ± 0.0950	0.0128 ± 0.0003
	MMVAE [39]	18.9029 ± 0.2252	0.0132 ± 0.0006
	M3VAE	**19.1276 ± 0.2019**	**0.0126 ± 0.0007**

Best results are marked in boldface.

**TABLE IV:** Biomarker Imputation Comparison

Data input	Method	MAE (ml) ↓	MRE (%) ↓
Bio	GRU-D [29]	5.6471 ± 0.3067*	7.5235 ± 0.4493*
	LSTM-T [28]	4.4112 ± 0.2774*	6.3127 ± 0.3136*
	MinimalRNN [46]	3.8784 ± 0.3542	5.7082 ± 0.2875
	BiPro [26]	4.1207 ± 0.4553	5.9002 ± 0.2700*
	LMDP-Net (IRLSTM)	**3.8504 ± 0.2823**	**5.6359 ± 0.2021**
Bio+Dg+Gen	GRU-D [29]	5.5422 ± 0.3055*	7.3789 ± 0.2946*
	LSTM-T [28]	4.1287 ± 0.2454*	6.0151 ± 0.2717*
	MinimalRNN [46]	3.9373 ± 0.3163	5.7233 ± 0.2599
	BiPro [26]	4.0700 ± 0.4840	5.9741 ± 0.3026*
	LMDP-Net (IRLSTM)	**3.8368 ± 0.2881**	**5.6338 ± 0.2877**
MRI+Bio+Dg+Gen	CNN-BRNN [13]	5.6713 ± 0.3005*	7.5433 ± 0.2819*
PET+MRI+Bio+Dg+Gen	LMDP-Net (M3VAE+IRLSTM)	**3.9917 ± 0.3798**	**5.7774 ± 0.2511**

Best results are marked in boldface; * denotes a significant difference.

**TABLE V:** Impact of Different Components in IRLSTM

Data Input	Method	Acc ↑	Pre ↑	Rec ↑	mAUC ↑
Bio	LSTM	0.5375 ± 0.0109	0.5363 ± 0.0090	0.5716 ± 0.0070	0.7328 ± 0.0083
	LSTM-Mask	0.5502 ± 0.0165	0.5479 ± 0.0189	0.5783 ± 0.0220	0.7399 ± 0.0081
	IRLSTM (β=2π)	0.5581 ± 0.0109	0.5533 ± 0.0104	0.5779 ± 0.0118	0.7409 ± 0.0062
	IRLSTM (β=π)	**0.5612 ± 0.0116**	**0.5563 ± 0.0134**	**0.5818 ± 0.0097**	**0.7410 ± 0.0076**
Bio+Dg+Gen	LSTM	0.5796 ± 0.0163	0.5745 ± 0.0166	0.5976 ± 0.0144	0.7601 ± 0.0065
	LSTM-Mask	0.5846 ± 0.0070	0.5803 ± 0.0082	**0.6096 ± 0.0079**	0.7650 ± 0.0095
	IRLSTM (β=2π)	0.5877 ± 0.0127	**0.5857 ± 0.0155**	0.6042 ± 0.0123	**0.7661 ± 0.0094**
	IRLSTM (β=π)	**0.5886 ± 0.0223**	0.5853 ± 0.0224	0.6085 ± 0.0187	0.7656 ± 0.0059
PET+MRI+Bio+Dg+Gen	LSTM	0.6018 ± 0.0189	0.6070 ± 0.0167	0.6049 ± 0.0247	0.7763 ± 0.0173
	LSTM-Mask	0.6058 ± 0.0204	0.6239 ± 0.0400	0.6059 ± 0.0093	0.7763 ± 0.0155
	IRLSTM (β=2π)	0.6132 ± 0.0200	0.6168 ± 0.0148	**0.6309 ± 0.0219**	0.7839 ± 0.0173
	IRLSTM (β=π)	**0.6222 ± 0.0128**	**0.6338 ± 0.0138**	0.6295 ± 0.0160	**0.7899 ± 0.0130**

Best results are marked in boldface.

**TABLE VI:** Status Prediction Comparison

Data Input	Method	Acc ↑	Pre ↑	Rec ↑	mAUC ↑
Bio	GRU-D [29]	0.5495 ± 0.0170*	0.5470 ± 0.0177*	0.5723 ± 0.0150*	0.7351 ± 0.0097*
	LSTM-T [28]	0.5460 ± 0.0108*	0.5449 ± 0.0151*	0.5760 ± 0.0091*	0.7379 ± 0.0093*
	MinimalRNN [46]	0.5511 ± 0.0071*	0.5470 ± 0.0098*	0.5731 ± 0.0071*	0.7203 ± 0.0083*
	BiPro [26]	0.5574 ± 0.0123*	0.5595 ± 0.0077*	0.5674 ± 0.0172*	0.7407 ± 0.0127*
	LMDP-Net (IRLSTM)	**0.5612 ± 0.0116**	**0.5563 ± 0.0134**	**0.5818 ± 0.0097**	**0.7410 ± 0.0076**
Bio+Dg+Gen	GRU-D [29]	0.5750 ± 0.0122*	0.5722 ± 0.0069*	0.5934 ± 0.0128*	0.7632 ± 0.0095*
	LSTM-T [28]	0.5703 ± 0.0088*	0.5645 ± 0.0088*	0.5907 ± 0.0082*	0.7572 ± 0.0041*
	MinimalRNN [46]	0.5680 ± 0.0203*	0.5661 ± 0.0211*	0.5885 ± 0.0185*	0.7549 ± 0.0091*
	BiPro [26]	0.5847 ± 0.0166*	**0.5867 ± 0.0219** *	0.5876 ± 0.0113*	0.7650 ± 0.0087*
	LMDP-Net (IRLSTM)	**0.5886 ± 0.0223**	0.5853 ± 0.0224	**0.6085 ± 0.0187**	**0.7656 ± 0.0059**
MR1+Bio+Dg+Gen	CNN-BRNN [13]	0.5947 ± 0.0209*	0.5955 ± 0.0211*	0.6000 ± 0.0161*	0.7654 ± 0.0178*
PET+MRI+Bio+Dg+Gen	Concatenation+IRLSTM	0.5923 ± 0.0234*	0.6058 ± 0.0298*	0.5978 ± 0.0259*	0.7709 ± 0.0250*
	MMVAE [39]+IRLSTM	0.6031 ± 0.0226*	0.6060 ± 0.0201*	0.6093 ± 0.0277	0.7683 ± 0.0222*
	MVAE [38]+IRLSTM	0.6141 ± 0.0182	0.6188 ± 0.0130*	0.6181 ± 0.0197	0.7854 ± 0.0136
	LMDP-Net (M3VAE+IRLSTM)	**0.6222 ± 0.0128**	**0.6338 ± 0.0138**	**0.6295 ± 0.0160**	**0.7899 ± 0.0130**

Best results arc marked in boldface; * denotes a significant difference.

**TABLE VII:** Effect of Using the Information of Current and T-1 Past Visits

T-l years	Acc ↑	Pre ↑	Rec ↑	mAUC ↑
0	0.6222 ± 0.0128	0.6338 ± 0.0138	0.6295 ± 0.0160	0.7899 ± 0.0130
1	0.8438 ± 0.0126	0.8339 ± 0.0164	0.8016 ± 0.0190	0.9407 ± 0.0076
2	0.8711 ± 0.0150	0.8702 ± 0.0217	0.8315 ± 0.0229	0.9547 ± 0.0078
3	0.9060 ± 0.0090	0.9074 ± 0.0153	0.8780 ± 0.0080	0.9675 ± 0.0043
4	0.9166 ± 0.0063	0.9197 ± 0.0117	0.8912 ± 0.0043	0.9723 ± 0.0031

**TABLE VIII:** Effects of Using and Not Using Diagnoses At the Current Visit (The $T^{th}$ Visit)

	Acc ↑	Pre ↑	Rec ↑	mAUC ↑
Without	0.6222 ± 0.0128	0.6338 ± 0.0138	0.6295 ± 0.0160	0.7899 ± 0.0130
With	0.8348 ± 0.0124	0.8306 ± 0.0134	0.7855 ± 0.0156	0.9400 ± 0.0063

**TABLE IX:** Comparison Between Unimodal and Multimodal Neuroimaging Data in the Prediction of AD Progression (AE is the Autoencoder Model)

Data Input	Method	Acc ↑	Pre ↑	Rec ↑	mAUC ↑
MRI+Bio+Dg+Gen	AE+IRLSTM	0.5890 ± 0.0156	0.5941 ± 0.0171	0.5995 ± 0.0123	0.7645 ± 0.0125
MRI+PET+Bio+Dg+Gen	LMDP-Net	**0.6182 ± 0.0138**	**0.6303 ± 0.0162**	**0.6257 ± 0.0174**	**0.7867 ± 0.0145**
PET+Bio+Dg+Gen	AE+IRLSTM	0.6138 ± 0.0309	0.6229 ± 0.0372	0.6344 ± 0.0210	0.7776 ± 0.0227
MRI+PET+Bio+Dg+Gen	LMDP-Net	**0.6327 ± 0.0335**	**0.6594 ± 0.0217**	**0.6244 ± 0.0560**	**0.7942 ± 0.0301**

The significance of bold values denote best results.

**TABLE X:** Comparison on Different Hidden State Sizes

Hidden state size	Acc ↑	Pre ↑	Rec ↑	mAUC ↑
64	0.6139 ± 0.0169	0.6154 ± 0.0167	0.6262 ± 0.0145	0.7823 ± 0.0166
128	**0.6222 ± 0.0128**	**0.6338 ± 0.0138**	**0.6295 ± 0.0160**	**0.7899 ± 0.0130**
256	0.6169 ± 0.0155	0.6270 ± 0.0217	0.6151 ± 0.0076	0.7853 ± 0.0177

The significance of bold values denote best results.

## Footnotes

1 Data used in the preparation of this article were obtained from the Alzheimer’s Disease Neuroimaging Initiative (ADNI) database (adni.loni.usc.edu). The ADNI was launched in 2003 as a public-private partnership, led by Principal Investigator Michael W. Weiner, MD. The primary goal of ADNI has been to test whether serial magnetic resonance imaging (MRI), positron emission tomography (PET), other biological markers, and clinical and neuropsychological assessment can be combined to measure the progression of mild cognitive impairment (MCI) and early Alzheimer’s disease (AD).

2 [Online]. Available: https://www.fil.ion.ucl.ac.uk/spm/software/spm12/
